# Supplementary material for: Predicting the impact of sequence motifs on gene regulation using single-cell data
Source: Genome Biol. 2023 Aug 15;24:189. doi: 10.1186/s13059-023-03021-9 (PMC10426127; doi:10.1186/s13059-023-03021-9)
Supplement: Supplementary file 1 — Additional file 1: Table S1. Comparison of deep learning approaches for regulatory sequence signal prediction. Table S2. Assignment of cell types to categories for the human kidney dataset. Table S3. Assignment of cell types to categories for the Tabula Muris dataset. [file 13059_2023_3021_MOESM1_ESM.pdf]

Supplementary Tables

Table S1: Comparison of deep learning approaches for regulatory sequence signal prediction.

Method	Predicts	Model architecture	Input sequence length
Xpresso	Steady-state mRNA levels	CNN with multiple convolutional layers	10,500 bp
scBasset	scATAC-seq data	CNN with multiple convolutional layers	1,344 bp
Enformer	Genome-wide CAGE-seq and epigenetic signals	CNN with multiple convolutional & transformer layers	196,608 bp
Sei	Genome-wide epigenetic signals	CNN with multiple convolutional layers	4,096 bp
BPNet	Genome-wide base-resolution ChIP-nexus signal	CNN with multiple convolutional layers	1,000 bp
Scover (this study)	Pooled scRNA-seq or pooled scATAC-seq data	CNN with one convolutional layer	1,000 bp (expression) or 240 bp (accessibility)

Table S2: Assignment of cell types to categories for the human kidney dataset

**Cell type**	**Category**
Ascending vasa recta endothelium	Endothelium
B cell	Immune
CD4 T cell	Immune
CD8 T cell	Immune
cDC2	Immune
CNT/PC - proximal UB	Nephron epithelium
Cap mesenchyme	Nephron progenitor
Connecting tubule	Nephron epithelium
Descending vasa recta endothelium	Endothelium
Distal S shaped body	Nephron progenitor
Distal renal vesicle	Nephron progenitor
Endothelium	Endothelium
Epithelial progenitor cell	Nephron progenitor
Fibroblast 1	Stroma
Fibroblast 2	Stroma
Glomerular endothelium	Endothelium
Indistinct intercalated cell	Nephron epithelium
Innate like lymphocyte	Immune
Loop of Henle	Nephron epithelium
MNP-a/classical monocyte derived	Immune
MNP-b/non-classical monocyte derived	Immune
MNP-c/dendritic cell	Immune
MNP-d/Tissue macrophage	Immune
Macrophage 1	Immune
Macrophage 2	Immune
Mast cells	Immune
Medial S shaped body	Nephron progenitor
Megakaryocyte	Immune
Monocyte	Immune
Myofibroblast	Stroma
Myofibroblast 1	Stroma
Myofibroblast 2	Stroma
NK cell	Immune
NKT cell	Immune
Neutrophil	Immune
pDC	Immune
Pelvic epithelium	Nephron epithelium
Pelvic epithelium - distal UB	Nephron progenitor
Peritubular capillary endothelium 1	Endothelium
Peritubular capillary endothelium 2	Endothelium

Podocyte	Nephron epithelium
Principal cell	Nephron epithelium
Proliferating B cell	Immune
Proliferating NK cell	Immune
Proliferating cDC2	Immune
Proliferating cap mesenchyme	Nephron progenitor
Proliferating distal renal vesicle	Nephron progenitor
Proliferating fibroblast	Stroma
Proliferating macrophage	Immune
Proliferating monocyte	Immune
Proliferating myofibroblast	Stroma
Proliferating stroma progenitor	Stroma
Proximal S shaped body	Nephron progenitor
Proximal UB	Nephron progenitor
Proximal renal vesicle	Nephron progenitor
Proximal tubule	Nephron epithelium
Stroma progenitor	Stroma
Thick ascending limb of Loop of Henle	Nephron epithelium
Type A intercalated cell	Nephron epithelium
Type B intercalated cell	Nephron epithelium

Table S3: Assignment of cell types to categories for the Tabula Muris dataset

**Cell type**	**Category**
bladder basal cell of urothelium	epithelial
bladder bladder cell	epithelial
bladder mesenchymal cell	connective
brainmicroglia microglial cell	immune
brainneurons astrocyte of the cerebral cortex	macroglial
brainneurons brain pericyte	pericyte
brainneurons endothelial cell	endothelial
brainneurons neuron	neuronal
brainneurons oligodendrocyte	macroglial
brainneurons oligodendrocyte precursor cell	macroglial
colon enterocyte of epithelium of large intestine	epithelial
colon epithelial cell of large intestine	epithelial
colon large intestine goblet cell	epithelial
fat B cell	immune
fat endothelial cell	endothelial
fat granulocyte	immune
fat mesenchymal stem cell of adipose	connective
fat myeloid cell	immune
fat natural killer cell	immune
fat neutrophil	immune
fat T cell	immune
heart cardiac muscle cell	muscle
heart endocardial cell	endothelial
heart endothelial cell	endothelial
heart epicardial adipocyte	adipose
heart fibroblast	connective
heart leukocyte	immune
heart smooth muscle cell	muscle
kidney kidney tubule cell	epithelial
liver endothelial cell of hepatic sinusoid	endothelial
liver hepatocyte	epithelial
lung endothelial cell	endothelial
lung stromal cell	connective
lung type II pneumocyte	epithelial
mammary basal cell	epithelial
mammary luminal epithelial cell of mammary gland	epithelial
mammary stromal cell	connective
marrow B cell	immune
marrow Fraction A pre-pro B cell	immune
marrow granulocyte	immune
marrow hematopoietic stem cell	immune
marrow monocyte	immune
marrow natural killer cell	immune
marrow neutrophil	immune
marrow T cell	immune
muscle B cell	immune
muscle endothelial cell	endothelial
muscle mesenchymal stem cell	connective

muscle skeletal muscle satellite cell	muscle
muscle skeletal muscle satellite stem cell	muscle
pancreas pancreatic A cell	endocrine
pancreas pancreatic acinar cell	exocrine
pancreas pancreatic D cell	endocrine
pancreas pancreatic ductal cell	epithelial
pancreas pancreatic PP cell	endocrine
pancreas type B pancreatic cell	endocrine
skin basal cell of epidermis	epithelial
skin epidermal cell	epithelial
skin keratinocyte stem cell	epithelial
spleen B cell	immune
spleen T cell	immune
thymus T cell	immune
tongue basal cell of epidermis	epithelial
tongue keratinocyte	epithelial
trachea epithelial cell	epithelial
trachea leukocyte	immune
trachea stromal cell	connective
