# Supplementary material for: Predicting the impact of sequence motifs on gene regulation using single-cell data
Source: Genome Biol. 2023 Aug 15;24:189. doi: 10.1186/s13059-023-03021-9 (PMC10426127; doi:10.1186/s13059-023-03021-9)
Supplement: Supplementary file 2 — Additional file 2: Figure S1. Statistics of the different datasets after pooling. (a) Largest cell type fraction per pool after pooling for the different datasets. (b) Number of cell types per pool for the different pooled datasets. (c) Total times a cell is included in pools for cells in the different datasets. (d) Fraction of non-uniquely assigned cells in a pool for the different pooled datasets. Figure S2. Correlations, number of cell types, sparsity (fraction of genes that are zero) and gene-wise coefficient of variation for influence scores (LOO gene CV) for different pool sizes for the datasets considered in this manuscript. The gene-wise coefficient for variation for influence scores was calculated only using reproducibly found motif families. Figure S3. (a) Example motif alignments for the human kidney dataset. (b) Random selection of 20 motifs with high impact scores that do not significantly align to the CIS-BP database. Figure S4. Pearson R2 for the different experimental setups. Pearson R2 for the held-out tests are shown for the models trained on the (a) human kidney, (b) Tabula muris, and (c) human brain datasets respectively. Some of the experiments on the x-axis reflect perturbation experiments, where either all motifs that aligned back to the database were left out (no non-aligned motifs), the order of the nucleotides in each sequence was permuted (scrambled sequences), or the order of each prediction vector was permuted (permuted cell pools). Figure S5. Projection of the pools onto the space represented by the first two principal components of the influence score matrix for the human kidney dataset. Color shows the summed influence scores for different motif clusters. Figure S6. Pairwise Pearson correlations between aggregate motif influence scores across pools between each of the reproducibly found motif clusters. Figure S7. (a) Example motif alignments for the Tabula Muris dataset. (b) Examples of randomly selected motifs with high influence sco [file 13059_2023_3021_MOESM2_ESM.pdf]

Supplementary Figures

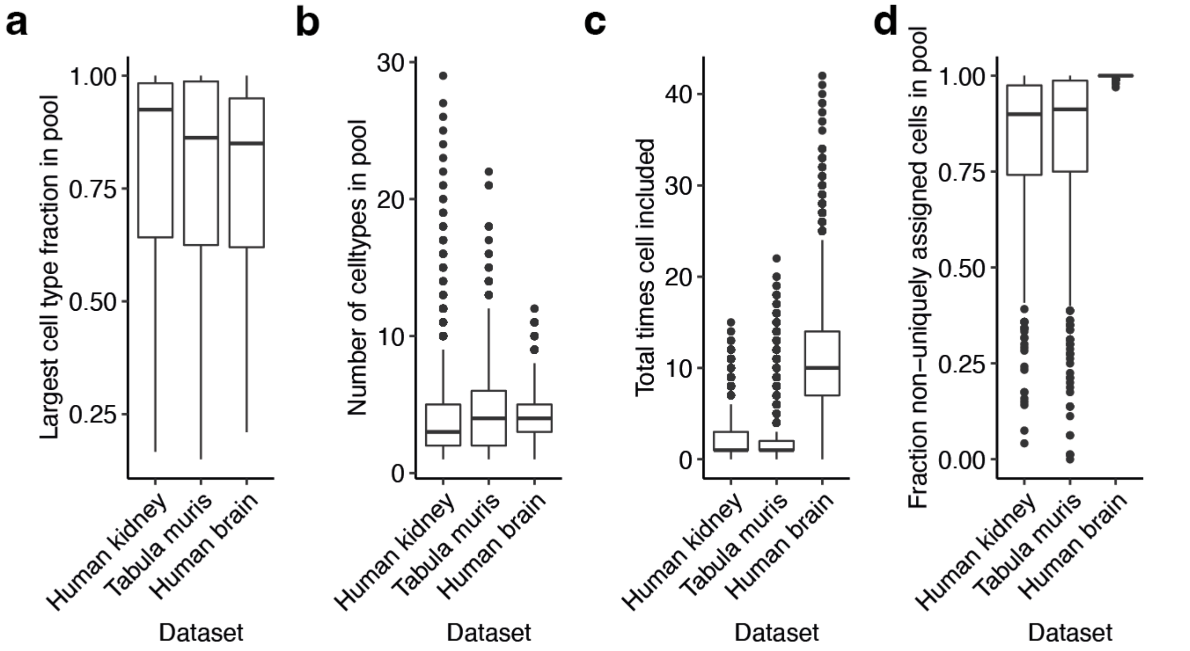

Figure S1: Statistics of the different datasets after pooling. (*a*) Largest cell type fraction per pool after pooling for the different datasets. (*b*) Number of cell types per pool for the different pooled datasets. (*c*) Total times a cell is included in pools for cells in the different datasets. (*d*) Fraction of non-uniquely assigned cells in a pool for the different pooled datasets.

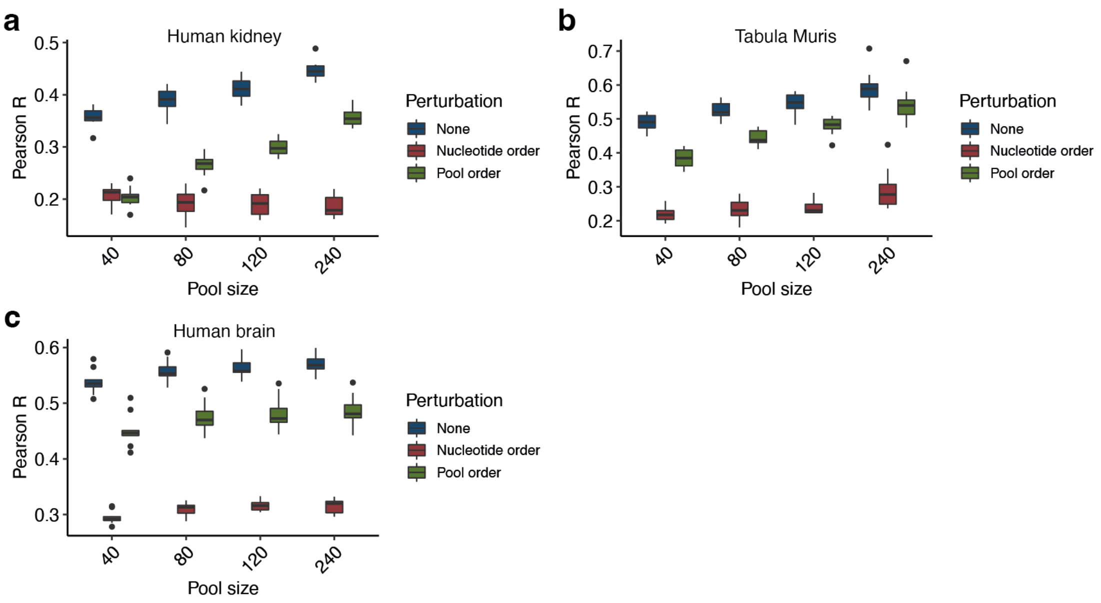

Figure S2: Correlations, number of cell types, sparsity (fraction of genes that are zero) and gene-wise coefficient of variation for influence scores (LOO gene CV) for different pool sizes for the datasets considered in this manuscript. The gene-wise coefficient for variation for influence scores was calculated only using reproducibly found motif families.

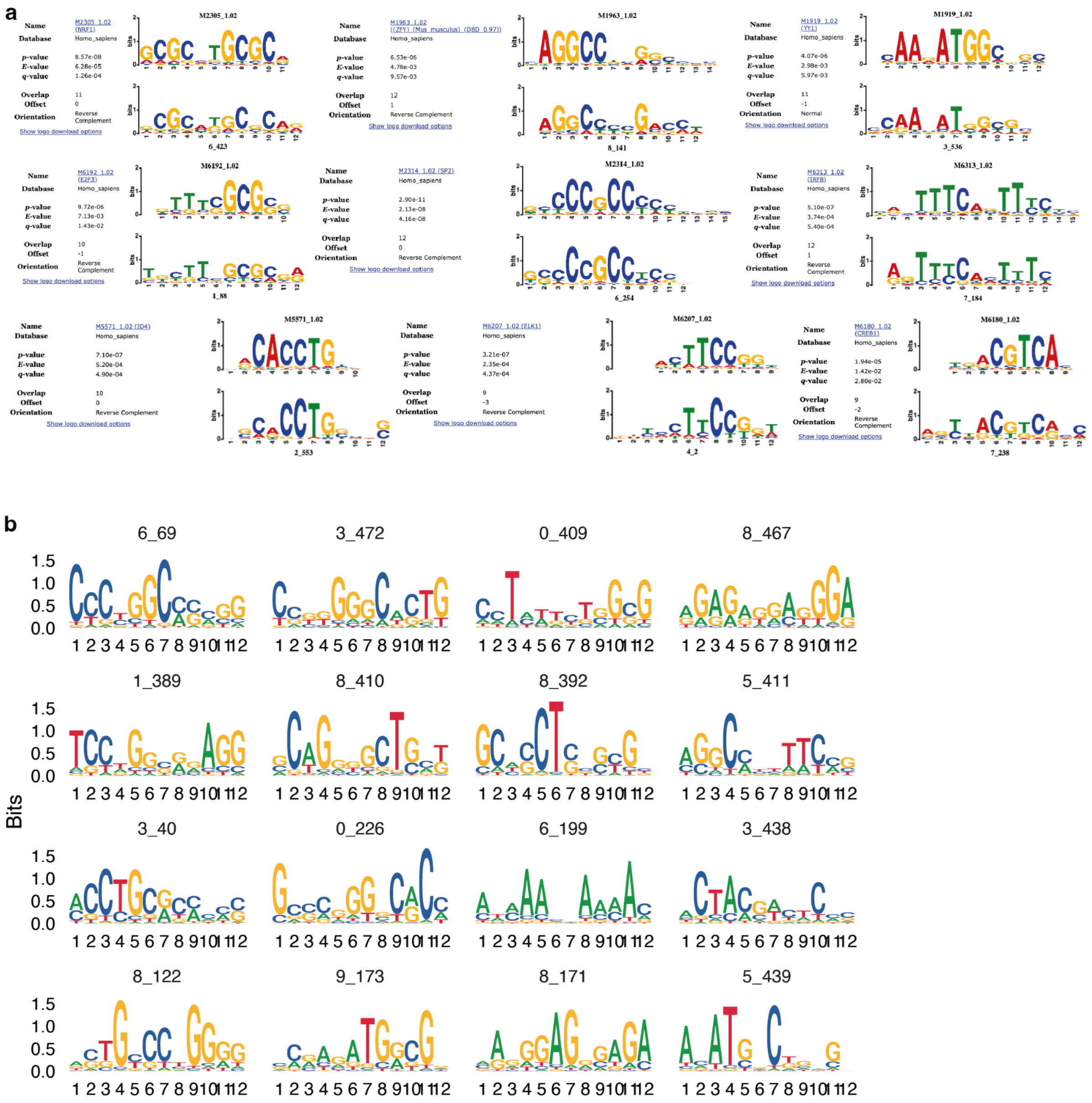

Figure S3: (*a*) Example motif alignments for the human kidney dataset. (*b*) Random selection of 20 motifs with high impact scores that do not significantly align to the CIS-BP database.

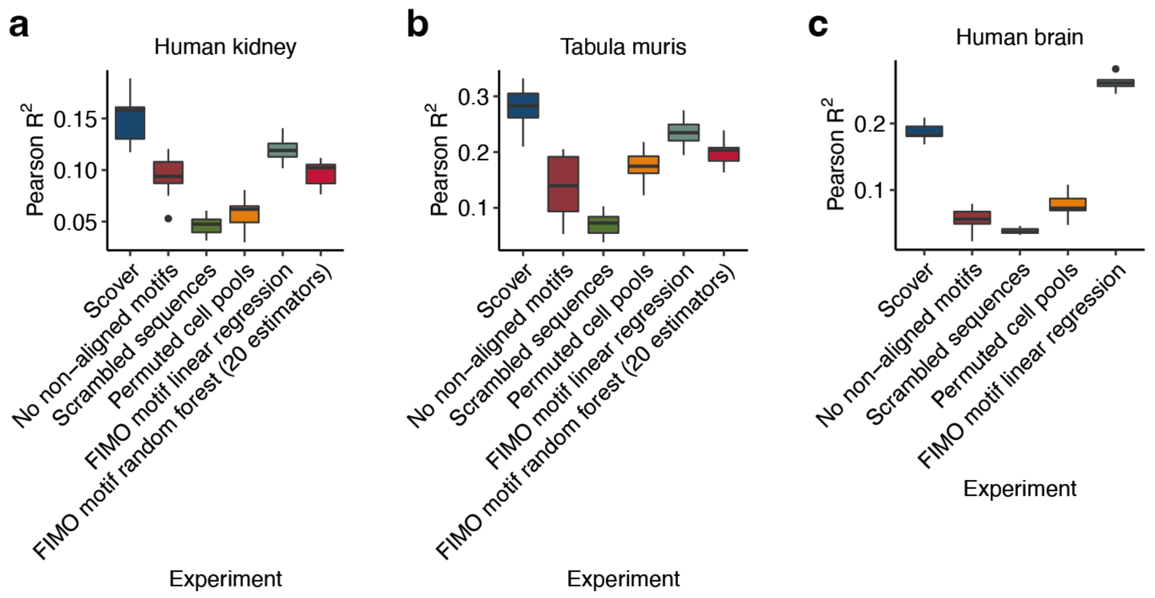

Figure S4: Pearson R^2 for the different experimental setups. Pearson R^2 for the held-out tests are shown for the models trained on the (*a*) human kidney, (*b*) Tabula muris, and (*c*) human brain datasets respectively. Some of the experiments on the x-axis reflect perturbation experiments, where either all motifs that aligned back to the database were left out (no non-aligned motifs), the order of the nucleotides in each sequence was permuted (scrambled sequences), or the order of each prediction vector was permuted (permuted cell pools).

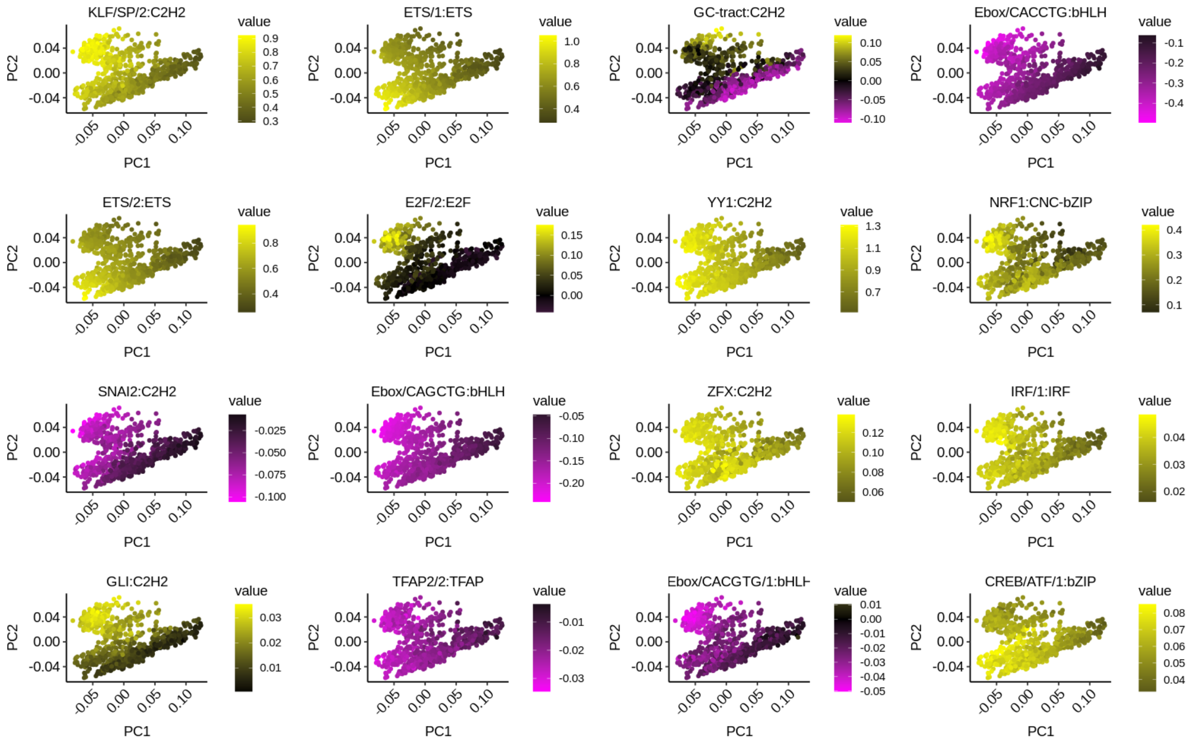

Figure S5: Projection of the pools onto the space represented by the first two principal components of the influence score matrix for the human kidney dataset. Color shows the summed influence scores for different motif clusters.

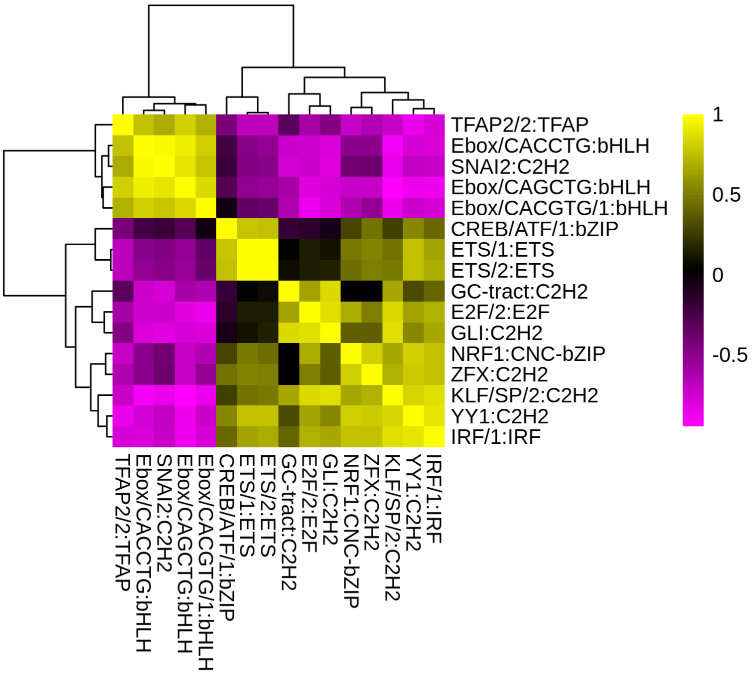

Figure S6: Pairwise Pearson correlations between aggregate motif influence scores across pools between each of the reproducibly found motif clusters.

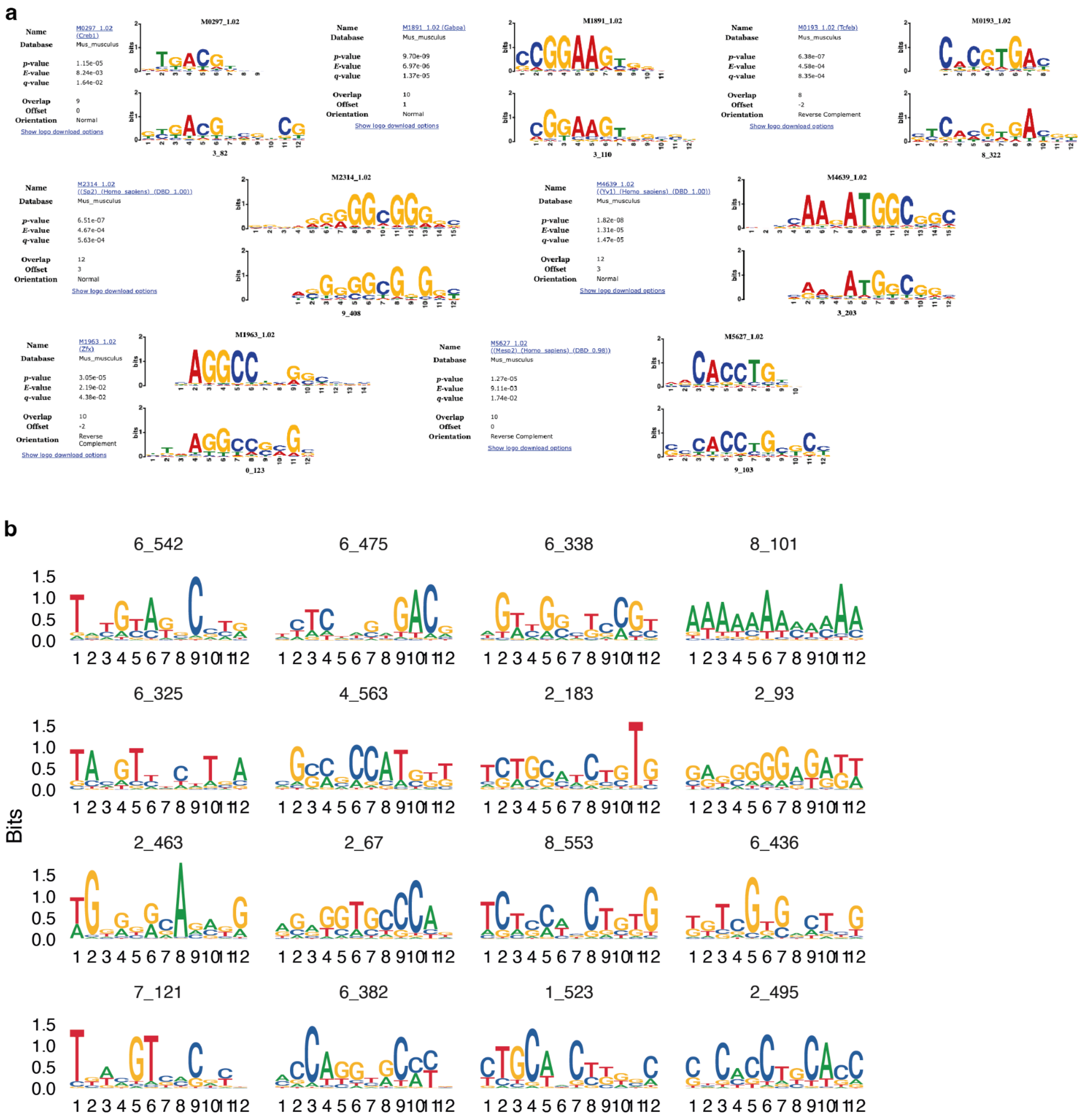

Figure S7: (*a*) Example motif alignments for the Tabula Muris dataset. (*b*) Examples of randomly selected motifs with high influence scores that did not align to CIS-BP. Motifs 2_183 and 2_495 are examples of E-box-like motifs.

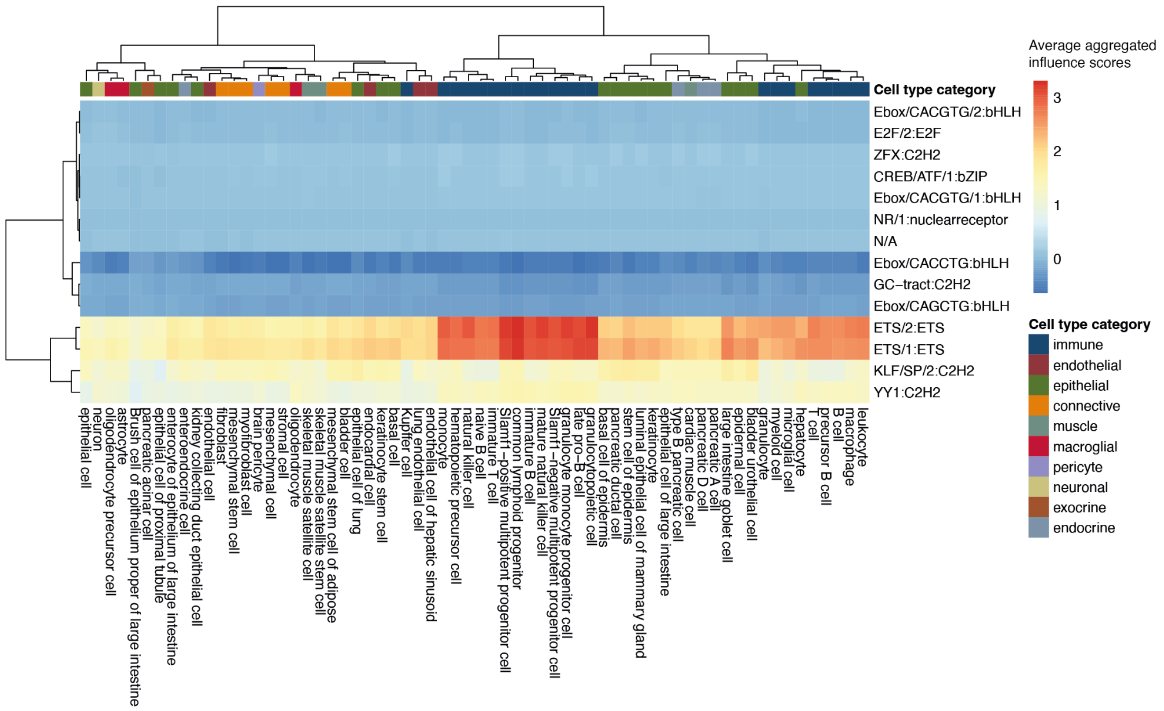

Figure S8: (*a*) Aggregate motif weights of motif clusters averaged in cell types for the Tabula Muris dataset. (*b*) Z-transformed aggregate motif weights of motif clusters averaged in cell types for the Tabula Muris dataset.

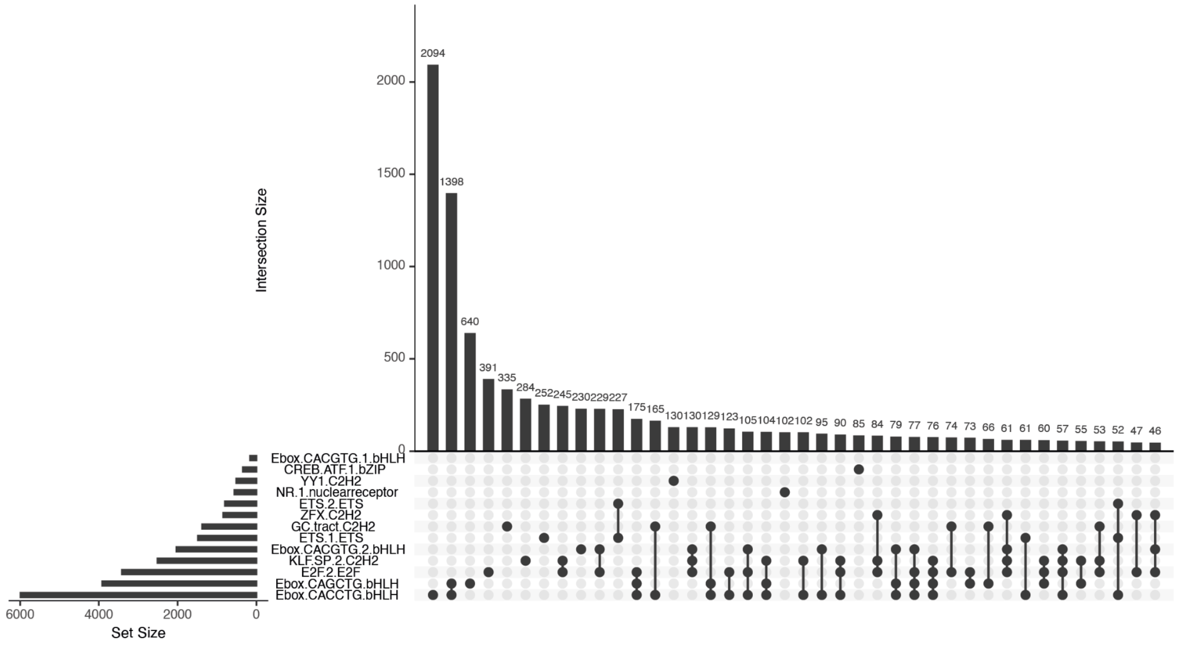

Figure S9: Co-occurring motif families in mouse promoters.

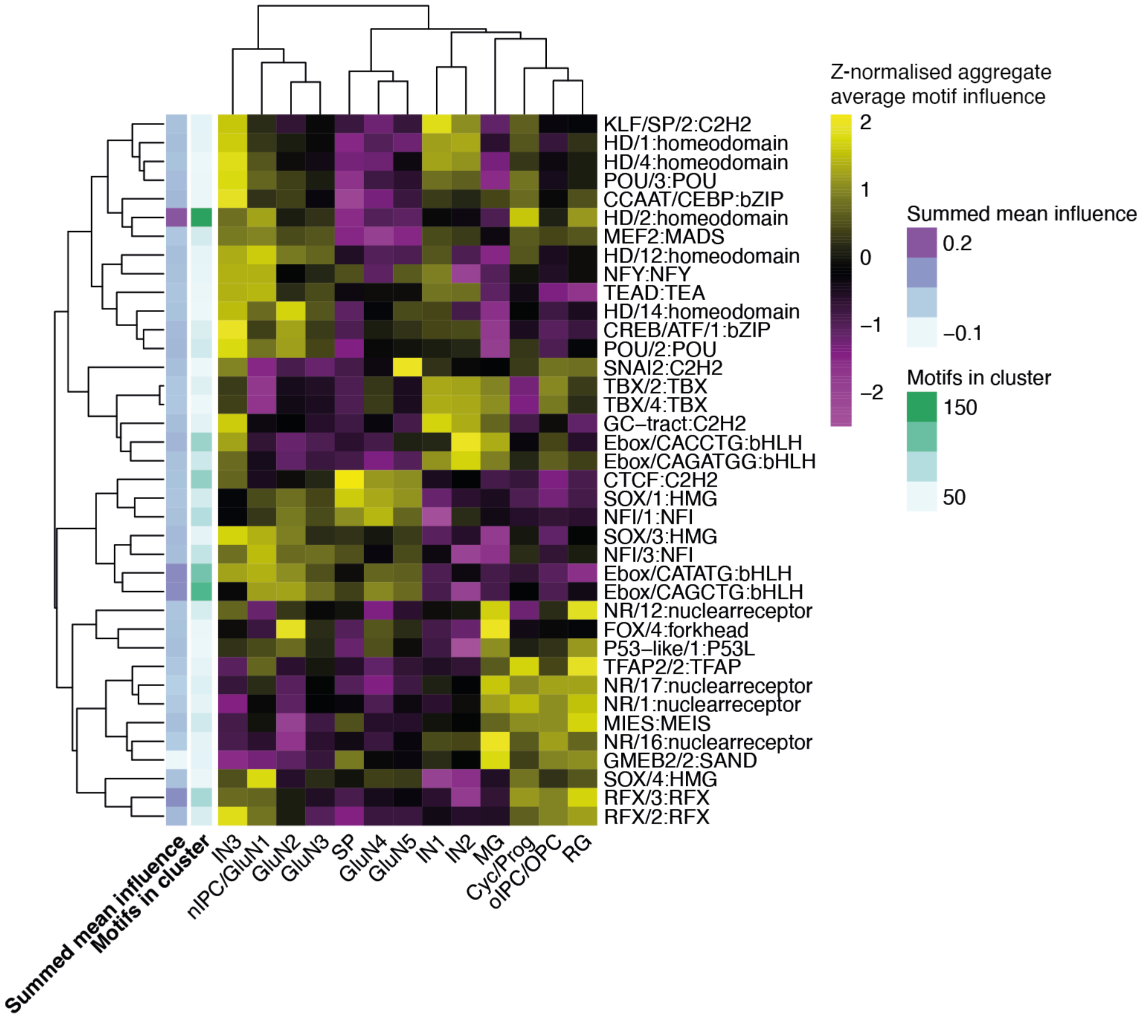

Figure S10: Z-transformed aggregate average motif influence scores for reproducibly found motif clusters for the model trained on the human brain dataset.

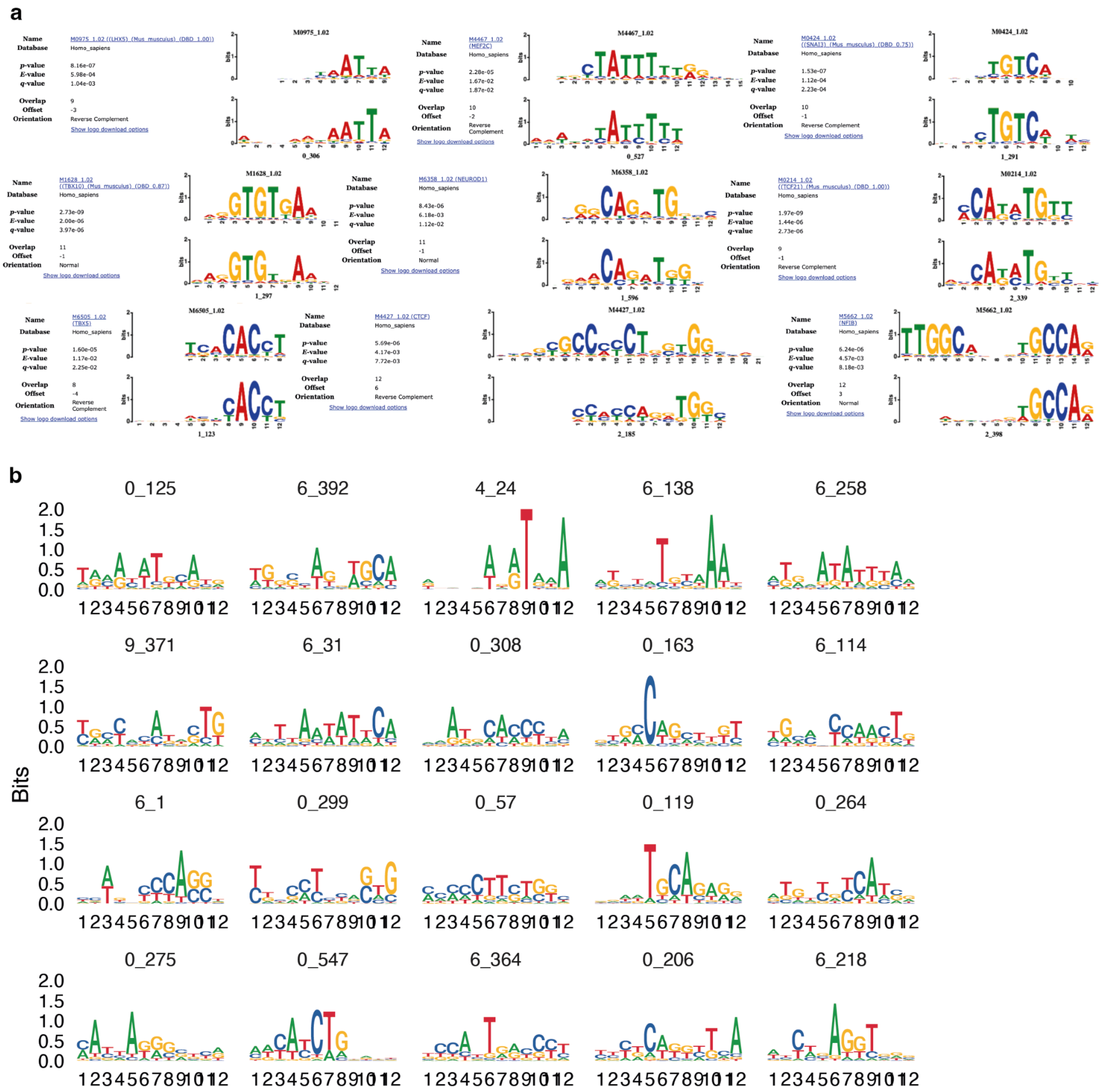

Figure S11: (*a*) Example motif alignments for the human brain dataset. (*b*) Examples of randomly selected motifs with high influence scores that did not align to CIS-BP for the human brain dataset.

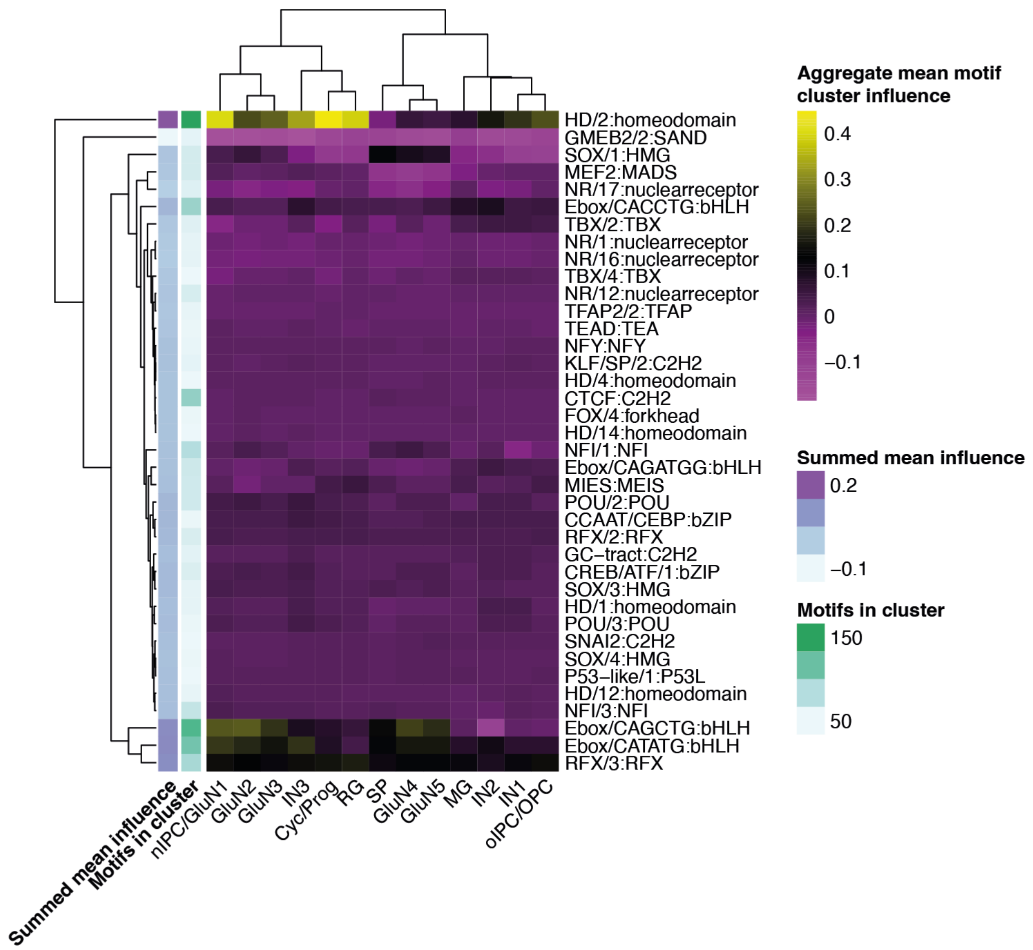

Figure S12: Aggregate mean motif cluster influence scores (averaged in cell types) from the model trained using the human brain data.
